# Clinical Significance of CUB and Sushi Multiple Domains 1 Inactivation in Head and Neck Squamous Cell Carcinoma

**DOI:** 10.3390/ijms19123996

**Published:** 2018-12-12

**Authors:** Ah Ra Jung, Young-Gyu Eun, Young Chan Lee, Joo Kyung Noh, Kee Hwan Kwon

**Affiliations:** 1 Department of Otolaryngology—Head & Neck Surgery, School of Medicine, Kyung Hee University, Seoul 02774, Korea; tlsdlcl24@naver.com (A.R.J.); ygeun@khu.ac.kr (Y.-G.E.); medchan@hanmail.net (Y.C.L.); 2 Department of Biomedical Science and Technology, Graduate School, Kyung Hee University, Seoul 02774, Korea; njookyung@naver.com; 3 Department of Otolaryngology—Head and Neck Surgery, Kangdong Sacred Heart Hospital, Hallym University College of Medicine, 150, Seongan-ro Gangdong-Gu, Seoul 134-701, Korea

**Keywords:** CSMD1, head and neck squamous cell carcinoma, prognosis, inactivation, gene signature

## Abstract

Although the genetic alteration of CUB and Sushi multiple domains 1 (CSMD1) is known to be associated with poor prognosis in several cancers, there is a lack of clinical relevance in head and neck cancer. The aim of this study was to offer insight into the clinical significance of CSMD1, utilizing a multimodal approach that leverages publicly available independent genome-wide expression datasets. CSMD1-related genes were found and analyzed to examine the clinical significance of CSMD1 inactivation in the HNSCC cohort of publicly available databases. We analyzed the frequency of somatic mutations, clinicopathologic characteristics, association with immunotherapy-related gene signatures, and the pathways of gene signatures. We found 363 CSMD1-related genes. The prognosis of the CSMD1-inactivated subgroup was poor. *FBXW7*, *HLA-A*, *MED1*, *NOTCH2*, *NOTCH3*, and *TP53* had higher mutation rates in the CSMD1-inactivated subgroups. The Interferon-gamma score and immune signature score were elevated in CSMD1-inactivated subgroups. We identified several CSMD1-related pathways, such as the phosphatidylinositol signaling system and inositol phosphate metabolism. Our study using three large and independent datasets suggests that CSMD1-related gene signatures are associated with the prognosis of HNSCC patients.

## 1. Introduction

Head and neck squamous cell carcinoma (HNSCC) arises from mucosa lining the paranasal sinuses, nasal cavities, oral cavity, nasopharynx, oropharynx, hypopharynx, and larynx [1]. Despite advances in our knowledge of its epidemiology, pathogenesis and treatment modalities, the survival rates of HNSCC have not improved over the past four decades, with 5-year survival rate remaining at 50% [2]. Since there is considerable clinicopathological heterogeneity among the tumors, a deeper understanding of pathogenesis of HNSCC is needed.

CUB and Sushi multiple domains 1 (CSMD1) is a large (~390 kDa) membrane-bound complement inhibitor [3]. It is composed of 14 N-terminal CUB domains separated by single complement control protein (CCP) domains and followed by 15 consecutive CCP domains. It has a single membrane-spanning domain at the C-terminus and a small cytoplasmic tail of 56 amino acids with a putative tyrosine phosphorylation site. Therefore, CSMD1 has been proposed to have an active role in cell cycle regulation and controlling apoptosis, for example, via the Smad pathway in melanoma cells [4]. CSMD1 is highly expressed in testis, cerebral cortex, cerebellum and brain white matter. A weaker expression was seen in breast, placenta and thyroid gland [3]. 

The CSMD1 gene occupies over 2 Mb in the short arm of chromosome 8 (8p23) [5]. Deletions of the short arm of chromosome 8 are some of the most common cytogenetic abnormalities seen in carcinomas and allelic imbalance or loss, indicative of hemizygous deletions, was observed in carcinomas of many different tissues [6]. CSMD1 is frequently shown to be deleted, mutated, or methylated in many cancers [7,8]. The loss of CSMD1 has been detected in many cases of HNSCC, lung cancer and breast cancer [9]. The results of previous study have shown association between 8p deletion or decreased CSMD1 expression and poor prognosis [8]. The analysis of gene mutation data derived from colon and breast cancers showed CSMD1 to be the most frequently mutated gene located on the p arm of chromosome 8 [8]. The clinical relevance of CSMD1 in HNSCC is limited to a small patient cohort, and study of the clinical relevance in a large cohort of HNSCC patients is needed.

The aims of this study were to find the gene signature related to CSMD1 and to investigate the clinical significance of CSMD1 utilizing a multimodal approach that leverages three large, publicly available, independent genome-wide expression datasets.

## 2. Results

### 2.1. Development and Clinical Significance of CSMD1-Related Gene Signature

**Figure 1:**
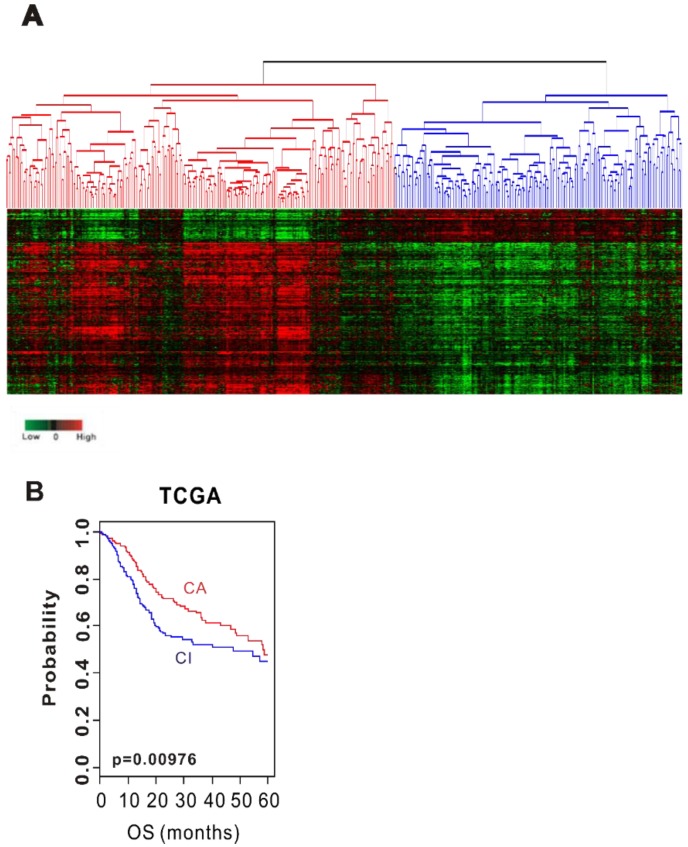
Stratification of HNSCC patients in the TCGA cohort with CSMD1 signature. (**A**) Hierarchical clustering of CSMD1 expression data in the TCGA cohort. (**B**) Kaplan–Meier plots of overall survival (OS) at 5 years of patients with HNSCC in the TCGA cohort.

We initially tested the prognostic value of CSMD1 per se, but did not show any significance (data not shown). For this reason, we hypothesized that several related genes would play a role together rather than the sole role of CSMD1 in HNSCC. We sought to find genes involved in the inactivation of CSMD1. This study used datasets from three independent cohorts. Table 1 details the pathological and clinical characteristics of the patients in all 3 cohorts. We identified genes whose expression is correlated with mRNA expression of CSMD1 in the training cohort (TCGA cohort) (*p* < 0.001 and Pearson correlation coefficient >0.4 or <−0.4). The expression of 363 genes was tightly associated with mRNA expression of CSMD1, and was selected as the CSMD1 signature (Table S1). Using the CSMD1 signature, we performed a hierarchical clustering analysis with the uncentered correlation coefficient as the measure of similarity and the complete linkage clustering method. Hierarchical clustering analysis of the gene expression data from the training data set (TCGA cohort, *n* = 513) revealed 2 distinct subgroups of HNSCC; CSMD1-activated (*n* = 294, CA: Red) subgroup and CSMD1-inactivated (*n* = 219, CI: Blue) subgroup (Figure 1A). The CA subgroup had significantly higher mRNA expression of CSMD1 than the CI subgroup (3.82 vs. 0.94, *p =* 3.7 × 10^−48^). The Kaplan–Meier plots and the log-rank test showed significantly different overall survival (OS) between two subgroups. The OS at 5 years was 52% in the CA subgroup and 46% in the CI subgroup. The OS of patients in the CI subgroup were significantly worse than those of patients in CA subgroup (*p* < 0.01; Figure 1B).

**Figure 2:**
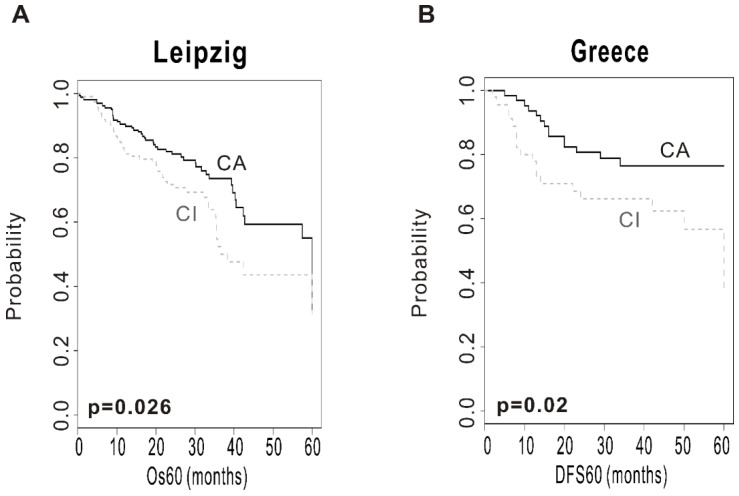
Construction of the prediction model and evaluation of predicted outcome. (**A**) Kaplan–Meier plots of overall survival (OS) at 5 years in the Leipzig cohort. (**B**) Kaplan–Meier plots of disease-free survival (DFS) at 5 years in the Greece cohort. Patients were stratified by CSMD1 signature. The differences between groups were significant, as indicated by the log-rank test.

To evaluate the robustness of the CSMD1 signature, validation was done in 2 independent test cohorts (Leipzig cohort, *n* = 270; Greece cohort, *n* = 109). The patients from the test cohorts were classified into CA and CI subgroups by prediction model based on BCCP. The Kaplan–Meier plots and the log-rank test showed significantly different prognosis between two subgroups. Kaplan–Meier plots and the log-rank test showed significant differences in OS in the Leipzig cohort (*p* = 0.026; Figure 2A) and disease-free survival (DFS) in the Greece cohort (*p* = 0.02; Figure 2B). The OS at 5 years was 49% in the CA subgroup and 38% in the CI subgroup in the Leipzig cohort. In addition, the DFS at 5 years was 80% in the CA subgroup and 40% in the CI subgroup in the Greece cohort. These results demonstrated the robustness of prognostic value of the CSMD1 signature. 

To evaluate the independent prognostic value of CSMD1 signature, univariate and multivariate Cox proportional hazards regression analyses were performed in the TCGA and Leipzig cohorts (*n* = 712), because of the available clinical data. In the univariate analysis, age (<60 years old vs. ≥60 years old), anatomic site (oropharynx vs. other site), T stage (T1 & T2 vs. T3 & T4) and the CSMD1 inactivation were significantly associated with overall survival. In the multivariate analysis, T stage and CSMD1 inactivation were independent prognostic factors in HNSCC [HR (95% CI), 1.97 (1.26–3.10); *p* = 0.003 and HR (95% CI), 1.42 (1.07–1.87); *p* = 0.012] (Table 2).

### 2.2. Association with CSMD1 Inactivation and Clinicopathologic Characteristics of HNSCC

To assess the association of CSMD1 inactivation with clinically recognized characteristics of HNSCC, subset analyses were performed in the TCGA and Leipzig cohorts. We assessed the association of the CSMD1 subgroup with tumor sites, human papillomavirus (HPV) status, gender, smoking status, regional lymph node (LN) metastasis and T stage (Figure S1, Table 3).

Comparing tumor site, patients in CA subgroup were comprised of 35.83% oral cavity cancer, 30.21% oropharynx cancer, 27.63% larynx cancer and 6.32% hypopharynx cancer, while 69.06% of the CI subgroup were classified to oral cavity cancer (*p* = 2.2 × 10^−16^). When comparing the HPV status in groups across CSMD1 signature, 81 of 321 patients in the CA subgroup (25.23%) were HPV (+) and 240 patients (74.76%) were HPV (−) status. Of 225 CI subgroup patients, 14 (6.22%) were HPV (+) and 211 (93.77%) were HPV (−). A clinical feature of the CI subgroup presented lower frequency of HPV (+) status than the CA subgroup (*p* = 1.57 × 10^−8^). When comparing gender in groups across CSMD1 signature, 77 of 431 patients of CA subgroup (17.86%) were female and 354 patients (82.13%) were male. Of 320 CI subgroup patients, 98 (30.62%) were female and 222 (69.31%) were male (*p* = 6.25 × 10^−5^). In addition, we also assessed association with smoking status and CSMD1 signature. 77 of the 424 CA subgroup patients (18.16%) were non-smokers and 347 patients (81.83%) were smokers. 81 of the 316 CI subgroup patients (25.63%) were non-smokers and 235 patients (74.36%) were smokers (*p* = 0.018). LN metastasis and T stage between CA and CI subgroup were not significantly different.

### 2.3. Relationship between CSMD1 Inactivation and Somatic Mutation

To investigate the co-occurrence of somatic mutation and CSMD1 inactivation in HNSCC, we analyzed somatic mutation data of patients in the TCGA cohort (*n* = 493). We selected the most frequently mutated genes from previous study of TCGA. We evaluated the frequency of somatic mutations in 30 genes associated with HNSCC. Among these genes, *FBXW7*, *HLA-A*, *MED1*, *NOTCH2*, *NOTCH3*, and *TP53* showed significantly higher mutation rates for patients in the CI than in the CA subgroup (Figure S2 and Table S2). *DICER1*, *KMT2D*, *MYH2*, *NEF2L2*, *NID2*, *NSD1*, *PIK3CA*, *PIK3R1*, *PTEN*, *RB1*, *RP1*, *SYNE1*, *SYNE2*, *TGFBR2* and *TRAF3* showed a higher frequency of mutation in the CA subgroup (Figure S2 and Table S2).

### 2.4. Relationship with Immunotherapy-Related Signature

**Figure 3:**
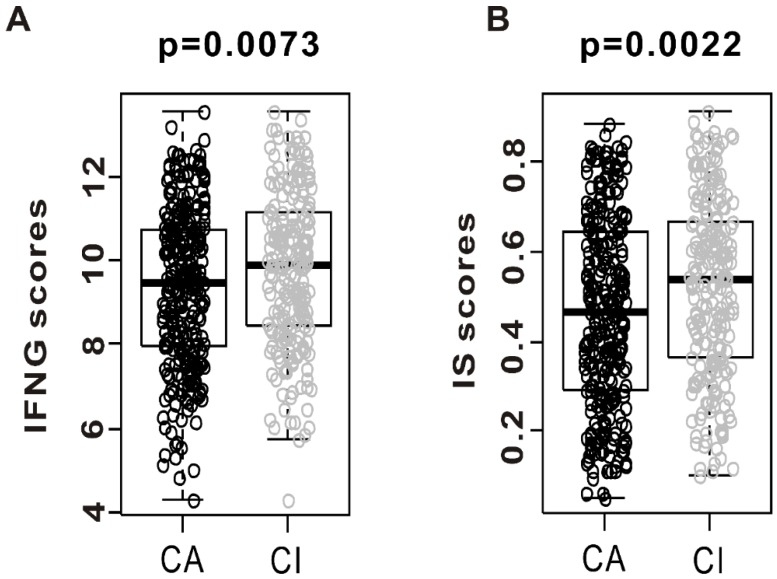
Evidence of immune activation in “CSMD1-activated” (CA) compared with “CSMD1-inactivated” (CI) subgroups. Gene expression level of interferon-gamma (IFNG) score and immune signature (IS) score were analyzed in both subgroups from the TCGA cohort. (**A**) Gene expression of interferon-gamma score was analyzed in CA and CI. (**B**) Immune signature score was also analyzed in both subgroups.

We sought to assess the association between CSMD1 inactivation and tumor immunity. We compared the interferon gamma (INFG) score and immune signature (IS) score between CA and CI. The IS scores were developed for identifying the responders to immunotherapy in a mouse model treated with anti-CTLA-4 antibodies. As shown in Figure 3A, INFG score was significantly elevated in CI when compared with the CA (*p* = 0.0073). We found that the IS scores were significantly higher in the CI than in the CA subgroup (Figure 3B, *p* = 0.0022). The patients with CSMD1 inactivation had higher INFG score and IS scores, suggesting that some of these might contribute to the response of immune checkpoint inhibitors such as anti-CTLA-4.

### 2.5. Biological Process and Pathway Analysis

Four GO biological process term enrichment analyses demonstrated that CSMD1 signature genes were characterized by synapse assembly, response to estradiol, positive regulation of synapse assembly and intrinsic apoptotic signaling pathway in response to DNA damage. Also, KEGG pathway function enrichment analysis for genes in CSMD1 signature was performed. Five KEGG pathways were found to be enriched, including inflammatory mediator regulation of TRP channels, phosphatidylinositol signaling system, glycine, serine and threonine metabolism, inositol phosphate metabolism, and small cell lung cancer (Table 4).

## 3. Discussion

HNSCC is the fourth most common gene alteration of CSMD1 in pancancer (17%), with a mutation of 5.2% and a deletion of 21% (Figure S3A). This suggests that gene alteration of CSMD1 might be an important genetic event for HNSCC. CSMD1 gene expression decreased regardless of copy number loss and somatic mutation (Figure S3B). CSMD1 inactivation is likely to occur for causes other than copy number loss or somatic mutation. Therefore, we sought to find the genes related to CSMD1 gene expression. We found the 363 genes (CSMD1 signature), and identified that CSMD1 inactivation was related to the prognosis of HNSCC. Loss of CSMD1 expression is associated with poor survival, and it is frequently deleted in breast cancer [10]. Deletions in CSMD1, a putative tumor suppressor implicated in diverse cancers, were found in a substantial fraction (26%) of oral squamous cell carcinoma patients [11]. Deletion and expression loss of this gene have been reported in association with poor survival, lymph node metastasis and advanced pathologic staging in several cancers [12].

Our results revealed that CSMD1 inactivation was significantly associated with low OS in training sets. In the validation set, CSMD1 inactivation was significantly associated with shorter OS and DFS at 5 years. This agrees with other studies that have reported an association between loss of the 8p23 region or CSMD1 and poor survival and high stage disease in prostate, bladder and head and neck cancer patients [10]. This suggests that CSMD1 may be a marker of advanced stage disease and disease recurrence [12]. In this study, the multivariate Cox regression model showed that CSMD1 inactivation and primary tumor size were independent factors for prognosis in HNSCC. This result agrees with a study in supraglottic cancer, where allelic loss at 8p23 appeared to be a statistically significant independent predictor of poor prognosis [13]. 

In our study, CSMD1 inactivation was accompanied by higher *FBXW7*, *HLA-A*, *MED1*, *NOTCH2*, *NOTCH3*, and *TP53* mutation rates, which could be responsible for treatment-related heterogeneity. *FBXW7* and *TP53* are known to be tumor suppressors [14,15]. Also, *HLA-A*, *MED1*, *NOTCH2* and *NOTCH3* are related with cancer prognosis [16,17,18]. In our results, we only know that the frequencies of these mutations are related to CSMD1 inactivation, but these data will be the basis of future research.

Therapies targeting the immune checkpoint molecules PD-1 and PD-L1 have achieved remarkable clinical responses in multiple types of cancers, including HNSCC. Identification of biomarkers that predict clinical benefit to immune-based approaches is needed. We sought to assess the association of CSMD1 inactivation with immune activation, such as elevation of INFG and IS scores. The analysis revealed that the CSMD1 inactivation subgroup had a potential response to immunotherapy. The IS scores reliably reported responders to immunotherapy in a mouse model treated with anti-CTLA-4 antibodies [19]. Most importantly, IS scores can identify responder patients with melanoma after treatment with ipilimumab [20]. Recent studies have shown that IFNG-related genes (INFG score) were indicative of response to immunotherapy in many cancers [21]. These results showed the possibility of association of CSMD1 inactivation with immune activation.

The biological processes and pathways of CSMD1 signature genes are shown in Table 4. Astrid et al. found that the EGFR/ PI3K/AKT, p38 MAPK and SRC-FAK pathways were the most affected pathways, whereas the effect on the STAT pathway was moderate in breast cancer-related CSMD1 [22]. Our results identified several pathways that are known to be important for cancer and HNSCC pathogenesis: the phosphatidylinositol signaling system (*p* = 0.021) and inositol phosphate metabolism (*p* = 0.028). Another important signaling pathway in cancer, including HNSCC, is the PI3K–PTEN–AKT pathway; the class Ia PI3Ks, which are most frequently associated with cancer, are heterodimers coupled to receptor tyrosine kinases such as EGFR or adaptor molecules that may become active after receptor phosphorylation [23]. It has also been shown that the identified mutations caused increased kinase activity, as well as increased migration and invasion of cells transfected with these mutants [24]. ITPKA, PIP5K1B, PLCE1, and PI4K2A associated with the CSMD1 signature genes are thought to play a crucial role in the PI3K–PTEN–AKT pathway. Our data offers support for the involvement of CSMD1 in signaling pathways with receptor or co-receptor involved in the process of signal transduction.

## 4. Materials and Methods

### 4.1. Patient Datasets

All clinical and gene expression data were collected previously and are available from public databases. Gene expression data of The Cancer Genome Atlas (TCGA cohort, *n* = 513) was downloaded from the UCSC Cancer Genomics Browser (Available online: https://genome-cancer.ucsc.edu/). The data from the Institute for Medical Informatics, Statistics and Epidemiology (Leipzig cohort, GSE65858, *n* = 270) and AHEPA Hospital in Thessaloniki (Greece cohort, GSE27020, *n* = 109) were obtained from the National Center for Biotechnology Information (NCBI) Gene Expression Omnibus (GEO) database (Available online: http://www.ncbi.nlm.nih.gov/geo) and used as the test sets [25,26]. Gene expression data of the TCGA cohort were generated by Illumina HiSeq2000, the Leipzig cohort by Illumina HumanHT-12 V4.0 Expression Beadchip, and the Greece cohort by Affymetrix U133A Genechips. All gene expression data were standardized because of different platforms.

### 4.2. Identification of Gene Signature

The BRB-ArrayTools software program (Available online: http://brb.nci.nih.gov/BRB-ArrayTools/) was used for analysis of gene expression data [27]. To find CSMD1-related genes in HNSCC, we applied the approach to gene expression data from TCGA cohort. We identified CSMD1-related genes by using Pearson’s correlation between mRNA expression of CSMD1 and mRNA expression of each gene. Genes were selected if the *p*-value was less than 0.001 and the correlation coefficient was more than 0.4 or less than −0.4. Hierarchical clustering analysis was done using Cluster 3.0 program and a heatmap was generated using TreeView software programs [28].

### 4.3. Construction of Prediction Models and Validation in Test Cohorts

To test the ability of the gene expression signatures to predict the class of patients in an independent cohort, a previously developed model based on Bayesian compound covariate predictor (BCCP) was adopted [29]. Gene expression data in the training set (TCGA cohort) were combined to form a series of classifiers according to the BCCP algorithm and the robustness of the classifier was estimated by the misclassification rate determined during leave-one-out cross-validation (LOOCV) of the training set. Validation was sought in two independent patient groups (Leipzig and Greece cohorts).

### 4.4. Association with CSMD1 Signature and Clinicopathologic Characteristics of HNSCC

The clincopathologic characteristics, such as tumor sites, human papillomavirus (HPV) status, gender, smoking status, regional lymph node (LN) metastasis and T stage, were collected in the TCGA and Leipzig cohorts because of available data. These data were analyzed to assess the association with CSMD1 signature.

### 4.5. Biological Process and Pathway Analysis

The list of genes of CSMD1 signature was submitted to the DAVID bioinformatics resources 6.7 (the Database for Annotation, Visualization, and Integrated Discovery), to discover the gene ontology (GO) categories with significantly enriched gene numbers [30,31]. The default setting from the software was used to map the CSMD1 signature genes to the reference set of direct and indirect relationships. Next, relevant input to the gene list, such as the molecular networks and biological functions, were generated by the software algorithm. KEGG (Kyoto Encyclopedia of Genes and Genomes) is an effort to link genomic information with higher-order functional information by computerizing current knowledge of cellular processes and by standardizing gene annotations [32]. 

### 4.6. Immunotherapy-Related Gene Signatures

A six-gene signature of INFG-related genes (*CXCL9*, *CXCL10*, *IDO1*, *IFNG*, *HLA-DRA*, and *STAT1*) was previously identified in melanoma samples from the KEYNOTE-001 study [33]. The INFG score was calculated as the average of the normalized values of the six genes [21]. The immune signature (IS) score was developed using the 105 immune signature genes [19]. The IS score of TCGA HNSCC cohort was obtained from the data of previous study [19]. These scores were compared according to CSMD1 signature.

### 4.7. Statistical Analysis

To test the prognostic significance, only gene expression data with available survival data were used. Pearson correlation was used for correlation analysis. Standardization was obtained by subtracting the median of the gene from the value of each gene and dividing by the standard deviation. Kaplan–Meier plots were constructed and log-rank tests performed for comparing the survival. Independent *t*-test was used to compare the mRNA expression of CSMD1 between CA subgroup and CI subgroup. Univariate and multivariate Cox proportional hazards regression analysis were performed to evaluate independent prognostic factors associated with survival. Fisher’s exact test was used to investigate the association of CSMD1 inactivation with clinically recognized characteristics of HNSCC and to assess the frequency difference of somatic mutation. All other statistical analyses were performed in the R language environment (Available online: http://www.r-project.org).

## 5. Conclusions

Our study suggests that CSMD1 inactivation is associated with the poor prognosis of HNSCC patients. CSMD1 inactivation might be related to HPV and tumor immunity.

## Figures and Tables

**Table 1:** Patient demographics and clinical characteristics of the three independent cohorts.

	TCGA Cohort (*N* = 513)	Leipzig Cohort (*N* = 270)	Greece Cohort (*N* = 109)
Gender			
Male	370 (73.7%)	223 (82.6%)	104 (95.4%)
Female	132 (26.3%)	47 (17.4%)	5 (4.5%)
Age (mean ± SD)	60.9 ± 11.9	60.1 ± 10.0	63 ± 10.0
Anatomic site			
Oral cavity	301 (60.0%)	83 (30.7%)	NA
Oropharynx	79 (15.7%)	102 (37.8%)	NA
Larynx	113 (22.5%)	48 (17.8%)	NA
Hypopharynx	9 (1.8%)	33 (12.2%)	NA
others	0	4 (1.5%)	NA
Primary tumor			
T1	33 (6.8%)	35 (13.0%)	NA
T2	147 (30.2%)	80 (29.6%)	NA
T3	129 (26.5%)	58 (21.5%)	NA
T4	178 (36.6%)	97 (35.9%)	NA
Regional lymph node			
N0	238 (49.5%)	94 (34.8%)	NA
N1	79 (16.4%)	32 (11.9%)	NA
N2	155 (32.2%)	132 (48.9%)	NA
N3	9 (1.9%)	12 (4.4%)	NA
Stage			
I	20 (4.1%)	18 (6.7%)	12 (11.0%)
II	96 (19.6%)	37 (13.7%)	18 (16.5%)
III	101 (20.7%)	37 (13.7%)	36 (33.0%)
IV	272 (55.6%)	178 (65.9%)	43 (39.4%)
HPV status			
Positive	68 (19.9%)	60 (23.4%)	NA
Negative	274 (80.1%)	196 (76.6%)	NA
Tobacco use			
Never	114 (23.3%)	48 (17.8%)	1 (0.9%)
Yes	376 (76.7%)	222 (82.2%)	108 (99.0%)
Alcohol use			
Never	154 (42.1%)	31 (11.5%)	51 (46.7%)
Yes	212 (57.9%)	239 (88.5%)	58 (53.2%)
CSMD1 signature			
CSMD1-activated	294 (57.3%)	158 (58.5%)	63 (57.7%)
CSMD1-inactivated	219 (42.7%)	112 (41.4%)	46 (42.2%)

Abbreviations: TCGA, The Cancer Genome Atlas; HPV, Human papilloma virus; NA, not available.

**Table 2:** Univariate and multivariate Cox proportional hazard regression analysis of overall survival in the TCGA and Leipzig cohorts (*n* = 712).

Variables	Univariate		Multivariate	
	HR (95% CI)	*p*-Value	HR (95% CI)	*p*-Value
CSMD1 inactivation	1.59 (1.23–2.04)	0.00036 *	1.42 (1.07–1.87)	0.012 *
Gender (male)	0.80 (0.60–1.07)	0.14	0.94 (0.68–1.29)	0.72
Age (≥60 years old)	1.32 (1.02–1.71)	0.031 *	1.25 (0.95–1.64)	0.102
Smoking (YES)	1.01 (0.74–1.37)	0.94	1.02 (0.73–1.41)	0.904
Alcohol (YES)	0.86 (0.64–1.15)	0.32	0.99 (0.72–1.36)	0.966
Anatomic site (Oropharynx)	0.52 (0.30–0.90)	0.021 *	0.63 (0.36–1.10)	0.107
Primary tumor (T3 & 4)	1.71 (1.29–2.27)	0.00018 *	1.97 (1.26–3.10)	0.003 *
Regional lymph node (N+)	1.18 (0.91–1.52)	0.19	1.29 (0.93–1.79)	0.12
Stage (stage III & IV)	1.36 (0.98–1.90)	0.062	0.68 (0.37–1.24)	0.21

* *p* < 0.05.

**Table 3:** Association with CSMD1 inactivation and clinicopathologic characteristics of HNSCC.

	CSMD1-Activated Subgroup	CSMD1-Inactivated Subgroup	*p* Value
Tumor site			2.2 × 10^−16^
Oral cavity	35.83%	69.06%	
Oropharynx	30.21%	14.69%	
Larynx	27.63%	11.56%	
hypopharynx	6.32%	4.69%	
HPV status			1.57 × 10^−8^
HPV (+)	25.23%	6.22%	
HPV (−)	74.76%	93.77%	
Gender			6.25 × 10^−5^
Male	82.13%	69.31%	
Female	17.86%	30.62%	
Smoking			0.018
Smoker	81.83%	74.36%	
Non-smoker	18.16%	25.63%	
LN metastasis			0.07
Positive	58.39%	51.47%	
Negative	41.6%	48.53%	
T stage			0.228
T1 & T2	40.76%	35.83%	
T3 & T4	59.24%	64.17%	

All *p* value was obtained by Fisher’s exact test.

**Table 4:** GO term biological process enrichment results and significantly altered pathways in CSMD1 signature genes.

GO Terms Biological Process	Count	Molecules	*p*-Value
Synapse assembly	8	DSCAM, WNT7A, CEL, NRXN1, NRXN3, NLGN1, NRCAM, PCLO	8.9 × 10^−5^ *
Response to estradiol	8	DNMT3A, WNT7A, ARNT2, BMP7, CASP9, FOXA1, PTCH1, PTN	1.1 × 10^−3^ *
Positive regulation of synapse assembly	5	WNT7A, NRXN1, NRXN3, NLGN1, NTRK2	0.023 *
Intrinsic apoptotic signaling pathway in response to DNA damage	4	BAK1, BCL2, CASP9, SFN	0.049 *
**KEGG Pathway**	**Count**	**Molecules**	***p*-Value**
Inflammatory mediator regulation of TRP channels	6	F2RL1, ADCY5, MAP2K6, PIK3R3, PLA2G6, TRPV4	0.021 *
Phosphatidylinositol signaling system	6	DGKB, ITPKA, PI4K2A, PIP5K1B, PIK3R3, PLCE1	0.021 *
Glycine, serine and threonine metabolism	4	CHDH, CBS, GATM, PGAM1	0.025 *
Inositol phosphate metabolism	5	ISYNA1, ITPKA, PI4K2A, PIP5K1B, PLCE1	0.028 *
Small cell lung cancer	5	BCL2, CASP9, NOS2, PIK3R3, PIAS2	0.049 *
Ras signaling pathway	5	PLA2G6, RIN1, KSR2, VEGFC, HTRZ	0.08

* *p* < 0.05.

## Supplementary Materials

The following are available online at http://www.mdpi.com/1422-0067/19/12/3996/s1.
