# Supplementary material for: Clinical Significance of CUB and Sushi Multiple Domains 1 Inactivation in Head and Neck Squamous Cell Carcinoma
Source: Int J Mol Sci. 2018 Dec 12;19(12):3996. doi: 10.3390/ijms19123996 (PMC6321139; doi:10.3390/ijms19123996)
Supplement: Supplementary file 1 [file ijms-19-03996-s001.pdf]

Supplementary Materials

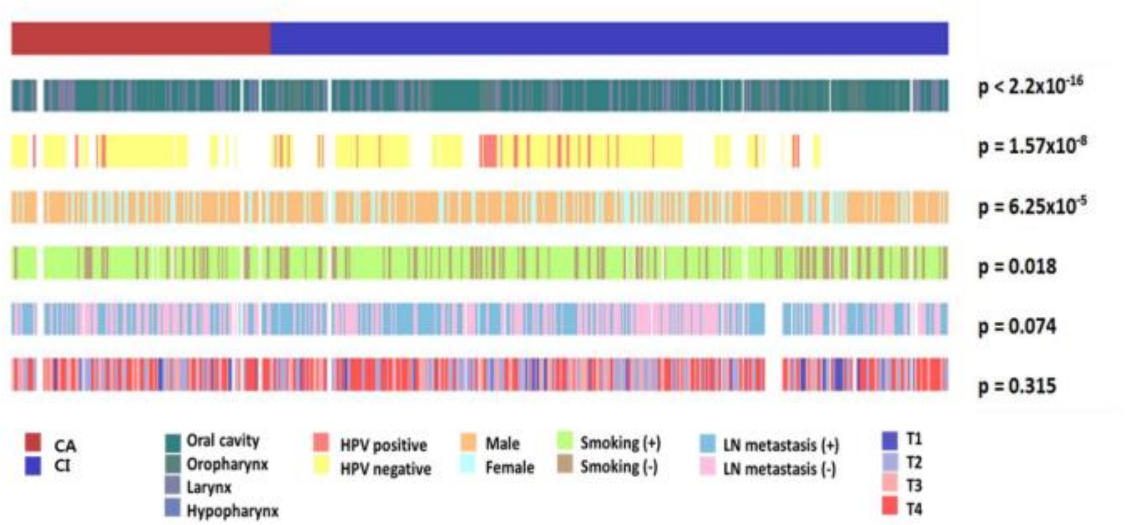

Supplementary FIGURE S1. Association with CSMD1 signature and other subtype of HNSCC patients in TCGA and Leipzig cohorts. Patients are displayed as columns, grouped by CSMD1 signature, tumor sites, human papillomavirus (HPV) status, gender, smoking status, regional lymph node (LN) metastasis and T stage.

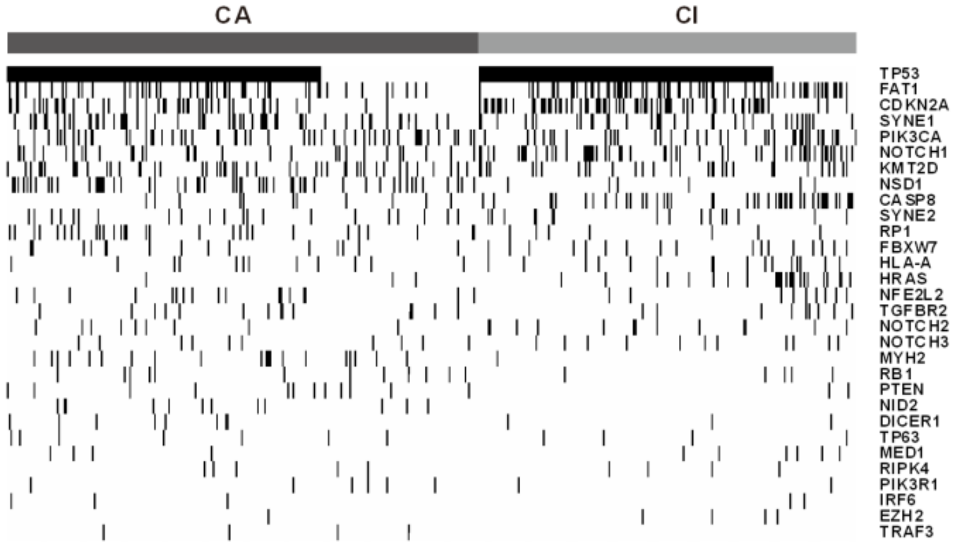

Supplementary FIGURE S2. Genetic changes in HNSCC cells according to CSMD1 signatures of patients in the TCGA cohorts. Samples are shown in columns and are grouped according to gene expression.

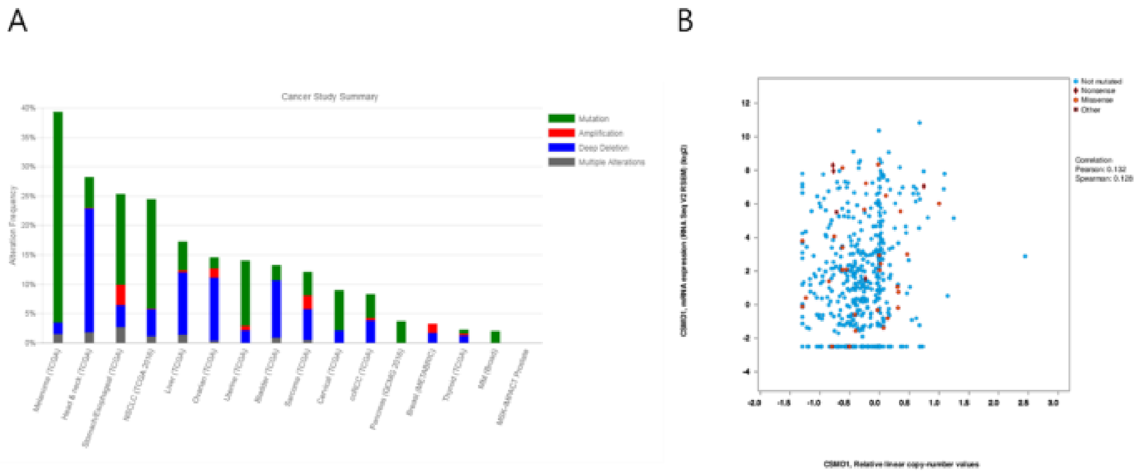

Supplementary FIGURE S3. *CSMD1* inactivation in HNSCC. (A) Cross-cancer summary of *CSMD1* alteration in 15 cancers by using genome copy number data obtained from The TCGA cohort. HNSCC is the second cancer showing *CSMD1* deep deletion (21%). The results shown here are based upon data generated by the TCGA Research Network (Available online: http://cancergenome.nih.gov/). (B) Scatter plots of *CSMD1* mRNA expression and copy number alteration in patients in the TCGA cohort.

Table S1. Genes associated with CSMD1.

TCGA RNA sequencing gene symbol	correlation coefficient with gene expression of CSMD1	p value of correlation with gene expression of CSMD1
ABCA13	0.443	4.70669E-26
ABCA17P	0.402	2.60822E-21
ABCA2	0.428	3.17546E-24
ABCC5	0.418	3.86414E-23
ACAP2	0.411	2.17204E-22
ACBD7	0.455	1.18115E-27
ACPL2	0.503	2.66918E-34
ACSS1	0.406	8.86937E-22
ACTB	-0.414	1.28719E-22
ACTN1	-0.410	3.05934E-22
ACTR1A	-0.454	1.67249E-27
ACVR2A	0.437	2.20163E-25
ADAM22	0.486	1.02248E-31
ADAMTS17	0.423	1.11484E-23
ADCY5	0.469	1.85709E-29
ADD3	0.428	2.62700E-24
AFF2	0.424	7.55193E-24
AKNAD1	-0.420	2.22453E-23
ALDH1A1	0.418	3.63025E-23
ALDH5A1	0.420	2.28757E-23
ANKRD20B	0.423	1.09640E-23
ANKRD6	0.447	1.26980E-26
AP3M2	0.406	7.97296E-22
ARHGAP24	0.478	1.31740E-30
ARHGEF19	0.415	9.99321E-23
ARNT2	0.499	1.23785E-33
ARSG	0.449	7.72168E-27
ATP6V1D	-0.431	1.23823E-24
BAK1	-0.462	1.71233E-28
BCAS3	0.438	1.61184E-25
BCL2	0.457	6.85370E-28
BEND5	0.436	3.22223E-25
BMP7	0.404	1.46083E-21
C10orf46	-0.410	2.97341E-22
C10orf75	0.409	4.27669E-22
C11orf92	0.441	8.62410E-26
C11orf93	0.419	3.08204E-23
C12orf34	0.500	9.25462E-34
C15orf56	0.421	1.96131E-23
C17orf28	0.406	9.74236E-22
C19orf33	-0.409	3.63645E-22

C1orf68	-0.411	2.57255E-22
C20orf141	-0.410	3.09662E-22
C20orf96	0.414	1.13042E-22
C21orf88	0.412	1.82932E-22
C2orf15	0.452	3.18798E-27
C2orf86	0.517	1.94872E-36
C3orf15	0.430	1.61629E-24
C3orf58	0.512	1.34476E-35
C3orf70	0.493	9.69093E-33
C7orf46	0.458	6.06283E-28
C9orf45	0.444	3.67138E-26
CAB39	-0.425	6.81201E-24
CACNA1D	0.411	2.66354E-22
CACNB4	0.410	3.31604E-22
CADPS2	0.406	9.73849E-22
CAP1	-0.402	2.21220E-21
CASP9	0.422	1.47252E-23
CAV1	-0.421	1.64726E-23
CBFA2T2	0.460	3.45038E-28
CBS	0.411	2.39094E-22
CCDC106	0.402	2.17702E-21
CCDC74A	0.423	1.20568E-23
CCDC74B	0.405	1.11471E-21
CCM2	-0.405	1.01399E-21
CDSN	-0.405	1.18287E-21
CEL	0.496	3.00385E-33
CENPC1	0.416	6.36291E-23
CEP68	0.440	1.04484E-25
CEP70	0.477	1.42619E-30
CHDH	0.478	1.34391E-30
CLDN3	0.446	2.06233E-26
CLGN	0.484	1.55065E-31
COCH	0.436	3.49557E-25
CPA4	-0.400	3.84645E-21
CRB2	0.407	6.94823E-22
CREB3L4	0.429	2.10708E-24
CTTNBP2	0.430	1.43738E-24
CYB5R1	-0.433	6.84153E-25
CYP26A1	0.454	1.76060E-27
CYP4X1	0.416	6.29178E-23
DAGLA	0.402	2.24421E-21
DCAKD	0.407	7.66167E-22
DEPDC5	0.410	2.86464E-22
DGKB	0.454	1.87947E-27
DHRS7	-0.480	5.30094E-31
DHX35	0.404	1.56053E-21
DMRTA2	0.435	3.92332E-25

DNAH7	0.437	2.69910E-25
DNAJC22	0.416	6.05947E-23
DNAJC27	0.417	4.76295E-23
DNMT3A	0.493	8.08893E-33
DSCAM	-0.424	9.17043E-24
ECE1	0.416	6.24073E-23
EIF2AK3	0.440	9.22415E-26
ENPP5	0.424	8.01856E-24
ENTPD4	0.446	2.05518E-26
EPB41L1	0.426	4.92422E-24
EPB41L4A	0.402	2.56637E-21
EPHA7	0.430	1.87024E-24
F2RL1	-0.403	1.82697E-21
F3	-0.406	8.25078E-22
FADS2	0.454	2.01021E-27
FAM117A	0.424	9.33538E-24
FAM161A	0.410	3.16937E-22
FAM171A1	0.430	1.76818E-24
FAM172A	0.499	1.13268E-33
FAM184B	0.461	2.17525E-28
FAM190A	0.437	2.67007E-25
FAM3B	0.460	2.66212E-28
FANCL	0.415	9.40304E-23
FBLIM1	-0.434	4.91223E-25
FHOD1	-0.476	2.21966E-30
FKBP6	0.455	1.18391E-27
FLJ13197	0.404	1.50405E-21
FLJ40852	0.408	4.85155E-22
FNDC8	0.426	5.10896E-24
FOXA1	0.483	2.47558E-31
FOXD3	0.421	1.73178E-23
FOXJ1	0.467	3.84525E-29
FOXP2	0.439	1.50156E-25
FOXRED2	0.469	1.90743E-29
FXYD5	-0.433	6.67140E-25
GALNT6	-0.429	2.03398E-24
GATM	0.408	4.92724E-22
GCNT2	0.475	3.10940E-30
GGA2	0.504	1.87022E-34
GGT7	0.459	4.02969E-28
GLS2	0.525	1.04313E-37
GOLGA7B	-0.441	8.20033E-26
GPLD1	0.452	3.45357E-27
GPR63	0.412	2.02318E-22
GPRC5B	0.457	7.13596E-28
GSR	0.448	9.71676E-27
HHAT	0.461	2.16730E-28

HLF	0.492	1.36365E-32
HS3ST4	0.405	1.26472E-21
HTR7	-0.474	3.85907E-30
HUNK	0.415	9.96207E-23
ICA1	0.401	2.76002E-21
ICK	0.400	3.75284E-21
IL17RB	0.460	3.48794E-28
IL34	0.403	1.90355E-21
ILDR1	0.401	2.93731E-21
IQCB1	0.409	3.65646E-22
ISM2	0.449	8.72183E-27
ISYNA1	0.408	5.88698E-22
ITPKA	0.423	1.14572E-23
KATNAL2	0.457	6.77335E-28
KCNG3	0.478	1.31311E-30
KCNH8	0.457	7.85580E-28
KCNK6	-0.401	3.14638E-21
KCTD3	0.405	1.11216E-21
KIAA0146	0.430	1.87720E-24
KIAA1609	-0.483	2.00351E-31
KIAA2022	0.434	5.10538E-25
KIFC3	-0.461	2.65112E-28
KLHL23	0.473	5.95253E-30
KRT1	-0.405	1.14789E-21
KRT14	-0.435	4.16158E-25
KRT16	-0.401	2.82816E-21
KRT19	0.406	8.61093E-22
KRT6B	-0.410	2.93571E-22
KSR2	0.424	7.49720E-24
L3MBTL4	0.440	1.07622E-25
LEF1	0.400	3.63996E-21
LHFPL1	0.425	5.82305E-24
LMO4	0.439	1.54840E-25
LOC100128640	0.428	3.07125E-24
LOC100190938	0.405	1.00043E-21
LOC284578	0.416	6.12704E-23
LOC400027	0.451	4.21519E-27
LOC541473	0.424	9.42488E-24
LOC730101	0.513	9.71828E-36
LONRF1	0.484	1.92692E-31
MAGI2	0.433	7.05105E-25
MAP2K6	0.433	8.11400E-25
MAP7D1	-0.463	1.36087E-28
MCCC1	0.441	7.07753E-26
MCF2L	0.520	6.33707E-37
MDFI	-0.469	1.91984E-29
MEIS1	0.420	2.25702E-23

MFSD4	0.470	1.50441E-29
MGAT3	0.426	4.82252E-24
MGC2889	0.404	1.35831E-21
MLLT6	0.415	8.69629E-23
MPPED2	0.407	6.22568E-22
MRAP2	0.528	3.60491E-38
MSI2	0.485	1.05762E-31
MSL2	0.424	7.39016E-24
MT2A	-0.499	1.02869E-33
MTUS1	0.447	1.26041E-26
MYB	0.451	5.06475E-27
MYLIP	0.400	3.46338E-21
MYO3A	0.537	9.96109E-40
MYO5C	0.504	1.90142E-34
NAALADL2	0.448	9.89203E-27
NCRNA00086	0.455	1.41795E-27
NFE2	-0.434	5.11372E-25
NINL	0.425	6.27732E-24
NIPAL4	-0.458	5.32174E-28
NLGN1	0.422	1.56753E-23
NOS2	0.452	3.78759E-27
NPPC	0.468	3.12242E-29
NR2F1	0.416	7.28589E-23
NR3C2	0.461	2.23917E-28
NRCAM	0.451	4.80865E-27
NRXN1	0.452	3.64561E-27
NRXN3	0.446	2.03405E-26
NSUN7	0.486	8.37387E-32
NTN3	0.431	1.19296E-24
NTRK2	0.486	9.83305E-32
NTS	0.498	1.46090E-33
OCA2	0.482	2.85049E-31
OTX1	0.460	3.46024E-28
OXGR1	0.413	1.45925E-22
P4HTM	0.470	1.46122E-29
PACRG	0.425	6.71582E-24
PAK7	0.406	7.76329E-22
PAN2	0.474	4.53847E-30
PAQR5	-0.406	8.09570E-22
PARM1	0.406	9.37471E-22
PATZ1	0.446	1.82457E-26
PBX1	0.489	3.06743E-32
PCDH19	0.436	3.42006E-25
PCLO	0.480	5.92955E-31
PDE5A	0.432	9.69552E-25
PGAM1	-0.427	4.08392E-24
PGPEP1	0.429	2.35759E-24

PHF8	0.442	6.19599E-26
PI4K2A	-0.437	2.41984E-25
PIAS2	0.422	1.41077E-23
PIK3R3	0.432	1.00620E-24
PIP5K1B	0.429	1.92240E-24
PLA2G6	0.401	2.75187E-21
PLCE1	0.417	4.76750E-23
PLEK2	-0.401	2.93032E-21
PLEKHG4	0.450	6.25826E-27
PLIN3	-0.413	1.32708E-22
PLK2	-0.434	5.30535E-25
PLSCR3	-0.458	5.33314E-28
PNP	-0.405	1.15190E-21
PODXL2	0.442	6.75341E-26
POGZ	0.408	4.81801E-22
PPM1D	0.408	4.83087E-22
PPM1H	0.431	1.26473E-24
PRKX	0.415	8.23153E-23
PRPSAP1	0.465	5.83890E-29
PSD3	0.419	2.98886E-23
PTCH1	0.437	2.19404E-25
PTN	0.442	5.31493E-26
PTPRS	0.436	2.87500E-25
PYGL	-0.436	3.19545E-25
RALGPS1	0.408	5.95358E-22
RANBP17	0.405	1.15227E-21
RASSF9	0.445	2.35892E-26
RBM11	0.407	7.55333E-22
REPIN1	0.467	4.21703E-29
RGS20	-0.528	3.14287E-38
RHBDL3	0.431	1.28390E-24
RHOD	-0.452	3.01096E-27
RIMKLA	0.442	5.39620E-26
RIN1	-0.429	2.27211E-24
RNF149	-0.405	1.25393E-21
RNF165	0.477	1.74290E-30
RNFT2	0.454	2.06491E-27
ROBO2	0.422	1.48273E-23
RPS6KA4	-0.460	3.32723E-28
RSBN1L	0.410	3.03021E-22
RTDR1	0.442	6.28182E-26
SALL2	0.402	2.38021E-21
SAMD12	0.510	2.52343E-35
SBK1	0.452	2.97837E-27
SCAI	0.448	1.18397E-26
SCML2	0.440	9.64280E-26
SERINC2	-0.415	8.13214E-23

SERPINI1	0.445	2.78284E-26
SETMAR	0.412	1.85906E-22
SFN	-0.446	2.15428E-26
SGEF	0.588	5.47877E-49
SLAIN1	0.432	9.37527E-25
SLC16A14	0.430	1.56507E-24
SLC1A1	0.403	1.78945E-21
SLC22A3	-0.411	2.26677E-22
SLC26A1	0.421	1.70756E-23
SLC26A5	0.409	4.09938E-22
SLC29A2	0.412	1.94620E-22
SLC6A11	-0.435	3.77763E-25
SLC7A2	0.461	2.10305E-28
SNX31	0.407	6.43344E-22
SOX21	0.444	3.19975E-26
SOX2OT	0.458	5.44721E-28
SP110	-0.414	1.26432E-22
SP9	0.457	6.58248E-28
SPAST	0.402	2.42684E-21
STK33	0.447	1.28815E-26
STOX1	0.432	8.50238E-25
STXBP6	0.454	2.12023E-27
SVIP	0.420	2.47361E-23
SYCP2	0.428	2.49988E-24
TACR2	0.415	7.94706E-23
TFDP2	0.404	1.57716E-21
THSD1	-0.406	9.30613E-22
TMEM116	0.410	2.86057E-22
TMEM150C	0.438	1.75640E-25
TMEM151B	0.426	4.78500E-24
TMEM56	0.400	3.60667E-21
TMPRSS2	0.426	4.89403E-24
TMSB15A	0.436	3.55499E-25
TMTC2	0.435	4.61690E-25
TMTC4	0.405	1.26387E-21
TNRC6C	0.471	1.11333E-29
TRIM2	0.429	2.25050E-24
TRIM45	0.418	4.36578E-23
TRPV4	0.457	7.54948E-28
TSGA10	0.418	4.26846E-23
TSPAN12	0.416	6.61472E-23
TSPYL2	0.413	1.47448E-22
TSPYL3	0.430	1.58706E-24
TTC21B	0.426	4.89919E-24
TTC23L	0.421	1.62401E-23
TTC28	0.454	1.97499E-27
TTLL11	-0.401	3.02576E-21

TUBA4A	-0.437	2.67664E-25
TUBB6	-0.478	1.10828E-30
TXNDC16	0.423	9.83024E-24
UBE2V1	-0.411	2.20685E-22
UGT2A1	0.449	8.96514E-27
UGT8	0.409	3.82415E-22
UNC119B	0.438	1.88994E-25
UPP1	-0.453	2.27641E-27
VEGFC	-0.446	1.75094E-26
VEZF1	0.429	2.10193E-24
VWA5B2	0.428	2.87673E-24
WDR49	0.486	8.44704E-32
WNK2	0.543	9.64573E-41
WNK3	0.479	9.52314E-31
WNT7A	-0.432	1.07714E-24
YBX2	0.427	3.39792E-24
ZBED3	0.419	3.08034E-23
ZBTB7C	0.422	1.34134E-23
ZDHHC2	0.461	2.23032E-28
ZFP14	0.506	9.31113E-35
ZNF10	0.413	1.33128E-22
ZNF189	0.402	2.14321E-21
ZNF214	0.415	9.06353E-23
ZNF227	0.447	1.55145E-26
ZNF238	0.452	3.64678E-27
ZNF248	0.409	4.01743E-22
ZNF396	0.421	1.93627E-23
ZNF420	0.411	2.27504E-22
ZNF507	0.428	2.95560E-24
ZNF519	0.433	7.69569E-25
ZNF541	0.419	3.27563E-23
ZNF546	0.412	2.08968E-22
ZNF566	0.467	3.39616E-29
ZNF599	0.411	2.70095E-22
ZNF606	0.401	3.25412E-21
ZNF620	0.441	8.16932E-26
ZNF667	0.417	5.94112E-23
ZNF711	0.472	8.88966E-30
ZNF780B	0.425	7.15934E-24
ZNF84	0.430	1.66787E-24

Table S2. Comparison of somatic mutation frequencies between CSMD1-activated and CSMD1-inactivated subgroups. P-values were obtained using the Fisher's exact test.

		CSMD1-activated	*CSMD1*-inactivated	Fisher's p value
CASP8	MUT	11	43	0.255
	WT	255	170	
CDKN2A	MUT	43	66	0.502
	WT	223	147	
DICER1	MUT	8	3	9.29E-04
	WT	258	210	
EZH2	MUT	1	4	1
	WT	265	209	
FAT1	MUT	48	62	0.417
	MUT	218	151	
FBXW7	WT	15	17	3.19E-03
	MUT	251	196	
HLA-A	WT	12	19	4.47E-03
	MUT	254	197	
HRAS	WT	3	24	0.303
	MUT	263	189	
IRF6	MUT	3	2	0.044
	WT	263	211	
KMT2D	MUT	46	30	5.19E-03
	WT	220	183	
MED1	MUT	4	6	0.01
	WT	262	207	
MYH2	MUT	20	1	3.57E-08
	MUT	246	212	
NEF2L2	WT	17	9	4.75E-04
	MUT	249	204	
NID2	WT	12	0	5.27E-07
	MUT	254	213	
NOTCH1	WT	31	51	0.144
	MUT	235	162	
NOTCH2	MUT	9	13	0.024
	WT	257	200	

NOTCH3	MUT	10	11	3.88E-03
	WT	256	202	
NSD1	MUT	52	6	4.96E-09
	WT	214	207	
PIK3CA	MUT	51	36	0.018
	MUT	215	177	
PIK3R1	WT	6	2	4.48E-03
	MUT	260	211	
PTEN	WT	12	2	9.89E-06
	MUT	254	211	
RB1	WT	12	5	1.00E-04
	MUT	254	208	
RIPK4	MUT	5	4	8.13E-03
	WT	261	209	
RP1	MUT	29	7	1.79E-06
	WT	237	206	
SYNE1	MUT	54	33	0.011
	WT	212	180	
SYNE2	MUT	21	16	9.19E-04
	MUT	245	197	
TGFBR2	WT	14	9	1.13-03
	MUT	252	204	
TP53	WT	177	166	2.92E-17
	MUT	89	47	
TP63	WT	7	4	1.15E-03
	MUT	259	209	
TRAF3	MUT	4	0	4.19E-04
	WT	262	213	
